# Impact of implementing the southampton physiotherapy post-operative screening tool (SPPOST)

**DOI:** 10.1186/2197-425X-3-S1-A554

**Published:** 2015-10-01

**Authors:** J Weblin, F Williams, DJ McWilliams

**Affiliations:** Therapy Services, UHB NHS Foundation Trust, Birmingham, United Kingdom

## Introduction

The benefit and necessity for prophylactic physiotherapy post operatively remains unclear [1]. Combined with an increased demand on resources, scores to identify patients who would most benefit are being increasingly used. A recent blinded observational evaluation of the SPPOST within our surgical physiotherapy service suggested it to be a potentially useful tool for identifying low risk patients who did not require physiotherapy intervention.

## Objectives

To formally introduce the SPPOST and analyse the impact of withdrawing physiotherapy intervention for low risk surgical patients on a liver surgical ward.

## Methods

All patients undergoing liver surgical procedures between 12^th^ July and 13^th^ August 2014 were included in the analysis. SPPOST scores were calculated on the first day post-operatively. For patients identified as low risk (Score < 10) post-operative care and mobilisation was led by the nursing staff with no physiotherapy involvement. Patients identified as high risk received standard physiotherapy input, including respiratory interventions and mobilisation as required. Primary outcome was development of post-operative pulmonary complications (PPC) according to the Brooks-Brunn criteria [2]. Secondary measures analysed were the number of low risk patients re-referred for physiotherapy, time to sit out of bed and time to walk >30 meters.

## Results

57 patients who had undergone liver surgery were included in the analysis, of which 24 (42%) were classified as low risk. None of the patients identified as low risk developed a PPC, although one low risk patient was re-referred to physiotherapy for upper limb exercises only. With regards to mobilisation, 20 (83%) of the low risk patients sat out of bed on day 1, whilst the remaining 4 (17%) did so on day 2, with no adverse events recorded. The median (IQR) time to walk 30 metres was 3 days [2-4]. Low risk patients spent on average 7.08 days in hospital, 1.47 less than the high risk group.

## Conclusions

Our results confirm the SPPOST as an effective post-operative screening tool, with no patients identified as low risk developing a PPC. For these low risk patients, mobilisation was safely led by nursing staff with none re-referred for either pulmonary physiotherapy or mobility assistance. However, recent enhanced recovery guidelines provide a target of walking patients on the first post-operative day, although very few patients achieved this goal in our low risk patient group. Further education for both patients and the nursing staff around 'early mobilisation' may assist patients to meet this target.

## References

[1] Pasquina P, Tramer MR, Granier J, Walder B (2006). Respiratory physiotherapy to prevent pulmonary complications after abdominal surgery. Chest.

[2] Brooks-Brunn JA (1997). Predictors of post-operative pulmonary complications following abdominal surgery. Chest.

